# Supplementary material for: Isolation and Characterization of Biocontrol Microbes for Development of Effective Microbial Consortia for Managing Rhizoctonia bataticola Root Rot of Cluster Bean Under Hot Arid Climatic Conditions
Source: Microorganisms. 2024 Nov 15;12(11):2331. doi: 10.3390/microorganisms12112331 (PMC11596626; doi:10.3390/microorganisms12112331)
Supplement: Supplementary file 1 [file microorganisms-12-02331-s001.zip › microorganisms-3311855-supplementary.pdf]

Supplementary table 1 A list of nutrient media used for isolation of microbes

S.N.	Media	Composition	Quantity
1.	Nutrient Agar Media	Peptone	5.0 g
		Beef extract	3.0 g
		NaCl	5.0 g
		Agar	15.0 g
		Distilled water	1000 mL
		pH	7.0
2.	T_3 Media	Tryptone	3.0 g
		Tryptose	2.0 g
		Yeast Extract	1.5 g
		$MnCl_2$	0.005 g
		Sodium Phosphate	0.05 g
		Agar	15.0 g
		Distilled water	1000 mL
		pH	6.8
3.	Jensen's Agar Media	Sucrose	20.0 g
		K_2HPO_4	1.0 g
		Mg_2SO_4	0.5 g
		NaCl	0.5 g
		Na_2MoO_4	0.001 g
		$FeSO_4$	0.01 g
		$CaCO_3$	2.0 g
		Agar	15.0 g
		Distilled water	1000 mL
		pH	7.0±0.2
4.	King's B Agar Media	Protease peptone	20.0 g
		K_2HPO_4	1.5 g
		$MgSO_4.7H_2O$	1.5 g
		Glycerol	10 mL
		Agar	15.0 g
		Distilled water	1000 mL
		pH	7.0±0.2
5.	Soli Extract Agar Media	Glucose	2.0 g
		Yeast Extract	1.0 g
		K_2HPO_4	0.5 g
		Soil extract*	100 mL
		Agar	15.0 g
		Distilled water	1000 mL
		pH	7.0±0.2
* Soil extract: 250 g Soil+ 1L Distilled water = Autoclave & filter			
6.	Trypticase Soya Agar Media	Trypticase	15.0 g
		Soya peptone	5.0 g

		NaCl	5.0 g
		Agar	15.0 g
		Distilled water	1000 mL
		pH	7.0±0.2
7.	R2A (Reasoner's 2A) Agar Media	Proteose peptone	0.5 g
		Casamino acids	0.5 g
		Yeast extract	0.5 g
		Dextrose	0.5 g
		Soluble starch	0.5 g
		K_2HPO_4	0.3 g
		$MgSO_4.7H_2O$	0.05 g
		Sodium pyruvate	0.3
		Agar	15.0 g
		Distilled water	1000 mL
		pH	7.0±0.2
8.	Semi-solid Nitrogen-Free bromothymol Malate Media NFb)	DL-malic acid	5.0 g
		KOH	4.0 g
		K_2HPO_4	0.5 g
		$MgSO_4.7H_2O$	0.1 g
		$CaCl_2$	0.01 g
		NaCl	0.02 g
		$Na_2MoO_4.2H_2O$	0.002 g
		$MnSO_4.2H_2O$	0.01 g
		$FeSO_4.2H_2O$	0.05 g
		Bromothymol blue	2 mL (0.5% alcoholic or dissolved in 0.2N KOH
		Agar	1.75 g
		Distilled water	1000 mL
		pH	7.0±0.2
9.	N-Free Okon, Albrecht, Burris Medium	Solution A:	
		DL-malic acid	5.0 g
		NaOH	3.0 g
		$MgSO_4.7H_2O$	0.2 g
		$CaCl_2$	0.02 g
		NaCl	0.1 g
		NH_4Cl	1.0 g
		Yeast extract	0.1 g
		$FeCl_3$	10 mg
		$Na_2MoO_4.2H_2O$	2.0 mg
		$MnSO_4$	2.1 mg
		H_3BO_3	2.8 mg
		$Cu(NO_3)_2.2H_2O$	0.04 mg
		$ZnSO_4.7H_2O$	0.24 mg
		Agar	18 g
		Distilled water	900 mL

		pH	6.8
		Solution B:	
		K_2HPO_4	6.0
		KH_2PO_4	4.0
		Distilled water	100 mL

Note: After autoclaving and cooling, the two solutions should be mixed. The medium pH is 6.8.

10.	Kenknight Agar medium	Dextrose	1.0 g
		KH_2PO_4	0.10 g
		$NaNO_3$	0.10 g
		KCl	0.10 g
		$MgSO_4.7H_2O$	0.10 g
		Agar	15 g
		Distilled water	1000 mL
		pH	7.0±0.2
11.	Pikovskaya	Glucose	10.0 g
		$(NH4)_2SO_4$	0.5 g
		$Ca(PO_4)_2$	5.0 g
		KCl	0.2 g
		$MgSO_4.7H_2O$	0.1g
		$MnSO_4$	Trace
		$FeSO_4$	Trace
		Yeast extract	0.5 g
		Agar	15
		Distilled water	1000 mL
		pH	7.0±0.2
12.	Potato Dextrose Agar Media	Potato infusion from 200 g pilled potato	1L
		Dextrose	20 g
		Agar	15 g
		pH	7.0±0.2
13.	Rose Bengal Agar Media	Enzymatic digest of soybean	5.0 g
		Dextrose	10.0 g
		KH_2PO_4	1.0 g
		$MgSO_4.7H_2O$	0.5 g
		Rose Bengal	0.05 g
		Chloramphenicol*	0.1 g
		Agar	15.0 g
		Distilled water	1000 mL
		pH	7.0±0.2

*Antibiotic should be added in autoclaved media after filter sterilization

14.	Czapek Dox Agar Media	Sucrose	30 g
		Sodium nitrate	2.0

	K_2HPO_4	1.0 g
	$MgSO_4.7H_2O$	0.50 g
	KCl	0.50 g
	$FeSO_4$	0.01 g
	Agar	15 g
	Distilled water	1000 mL
	pH	7.0±0.2

Supplementary Table 2. *In vitro* antagonistic assay of fungal isolates and *Rhizoctonia bataticola.*

**S.N.**	**Dual test**	**Diameter of T (cm)**	**Diameter of C (cm)**	**%Inhibition**
1	*Rhizoctonia bataticola* x 1F	1	8.5	88.24
2	*Rhizoctonia bataticola* x 2F	3	8.5	64.71
3	*Rhizoctonia bataticola* x 3F	3.8	8.5	55.29
4	*Rhizoctonia bataticola* x 4F	3.5	8.5	58.82
5	*Rhizoctonia bataticola* x 5F	1	8.5	88.24
6	*Rhizoctonia bataticola* x 6F	3.3	8.5	61.18
7	*Rhizoctonia bataticola* x 7F	2.8	8.5	67.06
8	*Rhizoctonia bataticola* x 8F	3.7	8.5	56.47
9	*Rhizoctonia bataticola* x 9F	3.1	8.5	63.53
10	*Rhizoctonia bataticola* x 10F	3.8	8.5	55.29
11	*Rhizoctonia bataticola* x 11F	3.8	8.5	55.29
12	*Rhizoctonia bataticola* x 12F	3.5	8.5	58.82
13	*Rhizoctonia bataticola* x 13F	3.6	8.5	57.65
14	*Rhizoctonia bataticola* x 14F	3.6	8.5	57.65
15	*Rhizoctonia bataticola* x 15F	3.4	8.5	60.00
17	*Rhizoctonia bataticola* x 37F	2	8.5	76.47
18	*Rhizoctonia bataticola* x 39F	3.8	8.5	55.29
19	*Rhizoctonia bataticola* x 41F	3.2	8.5	62.35
20	*Rhizoctonia bataticola* x 42F	1.5	8.5	82.35
21	*Rhizoctonia bataticola* x 43F	3.5	8.5	58.82
22	*Rhizoctonia bataticola* x 44F	3	8.5	64.71

*Data are the average of three replicates

Supplementary Table 3. *In vitro* antagonistic assay of bacterial isolates and *Rhizoctonia bataticola.*

S.N.	Dual test	Diameter of T (cm)	Diameter of C (cm)	% Inhibition
1	*Rhizoctonia bataticola* X 2B	3	8	62.5
2	*Rhizoctonia bataticola* X 3B	3	8	62.5
3	*Rhizoctonia bataticola* X 4B	7.5	8	6.25
4	*Rhizoctonia bataticola* X 5B	3	8	62.5
5	*Rhizoctonia bataticola* X 6B	6.8	8	15
6	*Rhizoctonia bataticola* X 7B	3.1	8	61.25
7	*Rhizoctonia bataticola* X9B	2	8	75
8	*Rhizoctonia bataticola* X 10B	2.8	8	65
9	*Rhizoctonia bataticola* X 11B	7.5	8	6.25
10	*Rhizoctonia bataticola* X 12B	3	8	62.5
11	*Rhizoctonia bataticola* X 15B	3.5	8	56.25
12	*Rhizoctonia bataticola* X 16B	2.8	8	65
13	*Rhizoctonia bataticola* X 17B	3	8	62.5
14	Rhizoctonia bataticolaX 18B	7.8	8	2.5
15	*Rhizoctonia bataticola* X 19B	3.1	8	61.25
16	*Rhizoctonia bataticola* X 25B	7.3	8	8.75
17	*Rhizoctonia bataticola* X 26B	6.5	8	18.75
18	*Rhizoctonia bataticola* X 27B	7.5	8	6.25
19	*Rhizoctonia bataticola* X 28B	6	8	25
20	*Rhizoctonia bataticola* X 29B	7.3	8	8.75
21	*Rhizoctonia bataticola* X 30B	3.5	8	56.25
22	*Rhizoctonia bataticola* X31B	3.5	8	56.25
23	*Rhizoctonia bataticola* X 32B	2	8	75
24	*Rhizoctonia bataticola* X 33B	7	8	12.5
25	*Rhizoctonia bataticola* X 34B	7	8	12.5
26	*Rhizoctonia bataticola* X 35B	7	8	12.5
27	*Rhizoctonia bataticola* X 36B	7	8	12.5
28	*Rhizoctonia bataticola* X 37B	7	8	12.5
29	*Rhizoctonia bataticola* X 38B	7.2	8	10
30	*Rhizoctonia bataticola* X 39B	7.2	8	10
31	*Rhizoctonia bataticola* X 40B	7.2	8	10
32	*Rhizoctonia bataticola* X 41B	7.2	8	10
33	*Rhizoctonia bataticola* X 48B	2.2	8	72.5
34	*Rhizoctonia bataticola* X 52B	4.5	8	43.75
35	*Rhizoctonia bataticola* X 68B	3	8	62.5
36	*Rhizoctonia bataticola* X 70B	7.5	8	6.25
37	*Rhizoctonia bataticola* X 71B	7.5	8	6.25
38	*Rhizoctonia bataticola* X 72B	3.5	8	56.25
39	*Rhizoctonia bataticola* X 73B	7	8	12.5
40	*Rhizoctonia bataticola* X 74B	7	8	12.5
41	*Rhizoctonia bataticola* X 75B	7	8	12.5
42	*Rhizoctonia bataticola* X 77B	7	8	12.5
43	*Rhizoctonia bataticola* X 78B	2	8	75
44	*Rhizoctonia bataticola* X 89B	3.5	8	56.25
45	*Rhizoctonia bataticola* X 91B	7	8	12.5
46	*Rhizoctonia bataticola* X 92B	7	8	12.5

47	*Rhizoctonia bataticola* X 93B	7	8	12.5
48	*Rhizoctonia bataticola* X 97B	7	8	12.5
49	*Rhizoctonia bataticola* X 98B	7	8	12.5
50	*Rhizoctonia bataticola* X 100B	7	8	12.5
51	*Rhizoctonia bataticola* X 101B	7	8	12.5
52	*Rhizoctonia bataticola* X 105B	7	8	12.5
53	*Rhizoctonia bataticola* X 106B	7	8	12.5
54	*Rhizoctonia bataticola* X 109B	3.3	8	58.75
55	*Rhizoctonia bataticola* X 110B	3.5	8	56.25
56	*Rhizoctonia bataticola* X 125B	7	8	12.5
57	*Rhizoctonia bataticola* X 131B	1	8	87.5
58	*Rhizoctonia bataticola* X 136B	6	8	25
59	*Rhizoctonia bataticola* X 139B	3.5	8	56.25
60	*Rhizoctonia bataticola* X 143B	2	8	75
61	*Rhizoctonia bataticola* X 155B	3.5	8	56.25
62	*Rhizoctonia bataticola* X 158B	2	8	75
63	*Rhizoctonia bataticola* X 159B	7.5	8	6.25
64	*Rhizoctonia bataticola* X 160B	7.5	8	6.25
65	*Rhizoctonia bataticola* X 164B	7.5	8	6.25
66	*Rhizoctonia bataticola* X 167B	7.5	8	6.25
67	*Rhizoctonia bataticola* X 169B	3	8	62.5
68	*Rhizoctonia bataticola* X 179B	2.1	8	73.75
69	*Rhizoctonia bataticola* X 182B	7.5	8	6.25
70	*Rhizoctonia bataticola* X 183B	7.5	8	6.25
71	*Rhizoctonia bataticola* X 185B	7.5	8	6.25
72	*Rhizoctonia bataticola* X 187B	7.5	8	6.25
73	*Rhizoctonia bataticola* X 189B	7.5	8	6.25
74	*Rhizoctonia bataticola* X 192B	2.7	8	66.25
75	*Rhizoctonia bataticola* X 193B	2	8	75
76	*Rhizoctonia bataticola* X 195B	3.1	8	61.25
77	*Rhizoctonia bataticola* X 196B	3.5	8	56.25
78	*Rhizoctonia bataticola* X 197B	3.2	8	60
79	*Rhizoctonia bataticola* X 200B	3.2	8	60
80	*Rhizoctonia bataticola* X 201B	3.2	8	60
81	*Rhizoctonia bataticola* X 205B	7	8	12.5
82	*Rhizoctonia bataticola* X 207B	3.2	8	60
83	*Rhizoctonia bataticola* X 209B	3.2	8	60
84	*Rhizoctonia bataticola* X 211B	3.5	8	56.25
85	*Rhizoctonia bataticola* X 214B	3.5	8	56.25
86	*Rhizoctonia bataticola* X 215B	3.6	8	55
87	*Rhizoctonia bataticola* X 216B	3.4	8	57.5
88	*Rhizoctonia bataticola* X 217B	3	8	62.5
89	*Rhizoctonia bataticola* X 218B	3.5	8	56.25
90	*Rhizoctonia bataticola* X 219B	3.4	8	57.5
91	*Rhizoctonia bataticola* X 220B	3.5	8	56.25
92	*Rhizoctonia bataticola* X 221B	2.8	8	65
93	*Rhizoctonia bataticola* X 223B	1.5	8	81.25
94	*Rhizoctonia bataticola* X 236B	1.5	8	81.25
95	*Rhizoctonia bataticola* X 239B	3.5	8	56.25

96	*Rhizoctonia bataticola* X 242B	1.8	8	77.5
97	*Rhizoctonia bataticola* X 248B	3.4	8	57.5
98	*Rhizoctonia bataticola* X 257B	3.5	8	56.25
99	*Rhizoctonia bataticola* X 261B	3.4	8	57.5
100	*Rhizoctonia bataticola* X 267B	3	8	62.5
101	*Rhizoctonia bataticola* X 271B	3.5	8	56.25
102	*Rhizoctonia bataticola* X 278B	3.4	8	57.5
103	*Rhizoctonia bataticola* X 284B	2.4	8	70
104	*Rhizoctonia bataticola* X 290B	3.5	8	56.25
105	*Rhizoctonia bataticola* X 304B	3.5	8	56.25
106	*Rhizoctonia bataticola* X 310B	2	8	75
107	*Rhizoctonia bataticola* X 325B	1.8	8	77.5
108	*Rhizoctonia bataticola* X 340B	3.5	8	56.25
109	*Rhizoctonia bataticola* X 341B	6	8	25
110	*Rhizoctonia bataticola* X 356B	3.2	8	60
111	*Rhizoctonia bataticola* X 370B	3.5	8	56.25
112	*Rhizoctonia bataticola* X 390B	1.8	8	77.5
113	*Rhizoctonia bataticola* X 391B	2.2	8	72.5
114	*Rhizoctonia bataticola* X 415B	3.3	8	58.75
115	*Rhizoctonia bataticola* X 439B	3.5	8	56.25
116	*Rhizoctonia bataticola* X 444B	3.5	8	56.25
117	*Rhizoctonia bataticola* X 445B	3.4	8	57.5
118	*Rhizoctonia bataticola* X 446B	3.3	8	58.75
119	*Rhizoctonia bataticola* X 460B	3.6	8	55
120	*Rhizoctonia bataticola* X 471B	3.2	8	60
121	*Rhizoctonia bataticola* X 473B	2	8	75
122	*Rhizoctonia bataticola* X 475B	2.7	8	66.25
123	*Rhizoctonia bataticola* X 477B	5	8	37.5
124	*Rhizoctonia bataticola* X 478B	6	8	25

*Data are the average of three replicates

Supplementary Table 4 . Morphological characterization of potent biocontrol bacterial isolates

Sl. N.		**Bacterial Isolates**		
		236B	223B	131B
1	Size	medium	large	small
2	Shape	rod	rod	rod
3	Margin	Irregular	lobate	undulate
4	Opacity	opaque	opaque	translucent
5	Elevation	flat	raised	convex
6	Texture	dry, rough	dry rough	mucoid
7	Pigmentation	Creamy	white	Greenish yellow

Supplementary Table 5. Physiological and biochemical characterization of potent biocontrol bacterial isolates

Sl.N.	Source	Bacterial Isolates		
		**236B**	**223B**	**131B**
1.	Lactose	-	-	+
2.	Xylose	+	-	+
3.	Maltose	-	-	+
4.	Fructose	-	-	+
5.	Dextrose	+	+	+
6.	Galactose	+	-	+
7.	Raffinose	-	-	+
8.	Trehalose	-	-	+
9.	Melibiose	+	-	+
10.	Sucrose	-	-	+
11.	L-Arabinose	+	-	+
12.	Mannose	+	-	+
13.	Inulin	-	+	-
14.	Sodium gluconate	-	+	-
15.	Glycerol	-	+	-
16.	Salicin	-	+	-
17.	Dulcitol	-	-	-
18.	Inositol	-	+	-
19.	Sorbitol	+	+	-
20.	Mannitol	-	-	-
21.	Adonitol	-	-	+
22.	Arabitol	-	-	-
23.	Erythritol	-	+	-
24.	α-Methyl-D-glucoside	-	+	-
25.	Rhamnose	-	+	+
26.	Cellobiose	-	+	-
27.	Melezitose	-	+	-
28.	α-Methyl-D-mannoside	-	+	-
29.	Xylitol	-	+	+
30.	D-Arabinose	-	-	-
31.	Sorbose	-	+	-
32.	Citrate utilization	+	+	+
33.	Malonate utilization	+	+	-
34.	ONPG	-	+	+
35.	Esculin hydrolysis	-	+	-
36.	Lysine utilization	-	-	-
37.	Ornithine utilization	-	-	-
38.	Urease	-	-	+
39.	Phenylalanine Deamination	-	-	-
40.	Nitrate reductase	-	-	+

41.	H2S production	-	-	-
42.	Oxidase	+	+	+
43.	Casein hydrolysis	+	+	-
44.	Catalase test	+	+	+
45.	KOH test	-	-	+
46.	Gram reaction	+	+	-

Supplementary Table 6. Microscopic and macroscopic characteristics of *Trichoderma afroharzianum* 5F

**Sl.N.**	**Characteristics**	**Fungal isolate**
		**5F**
1.	Spore shape	Globose to subglobose
2.	Color	Greenish
3.	Spore arrangement	In clusters at the tips of conidiophores
4.	Hyphae	Septate, hyaline, and smooth
5.	Fruiting bodies	Conidiophores branched with flask-shaped phialides
